# The complete chloroplast genome sequence of *Styrax calvescens* Perk. (Styracaceae)

**DOI:** 10.1080/23802359.2020.1715869

**Published:** 2020-01-22

**Authors:** Xiaogang Xu, Lili Tong, Yabo Wang, Chongli Xia, Yaoqin Zhang

**Affiliations:** a Co-Innovation Center for Sustainable Forestry in Southern China, College of Biology and the Environment, Key Laboratory of State Forestry and Grassland Administration on Subtropical Forest Biodiversity Conservation, Nanjing Forestry University, Nanjing, China;; b State Environmental Protection Scientific Observation and Research Station for Ecology and Environment of Wuyi Mountains;; c School of Horticulture and Landscape Architecture, Jinling Institute of Technology, Nanjing, China

**Keywords:** *Styrax calvescens*, phylogenomics, Styracaceae, chloroplast genome

## Abstract

*Styrax calvescens* Perk., is a nice tree, valued for its beauty and fragrance. Here, we characterized the complete chloroplast (cp) genome of *S. calvescens* using next-generation sequencing. The circular complete cp genome of *S. calvescens* is 157,951 bp in length, containing a large single-copy (LSC) region of 87,566 bp and a small single-copy (SSC) region of 18,289 bp. It comprises of 133 genes, including 8 rRNA genes, 37 tRNAs genes, and 88 protein-coding genes. The GC content of *S. calvescens* cp genome is 36.95%. The phylogenetic analysis suggests that *S. calvescens* is a sister species to *Styrax grandiflorus* Griffith in Styracaceae.

*Styrax calvescens* Perk. (Styracaceae), a nice tree with abundant white fragrant flowers blooming in late spring, is a multipurpose economic arbor species integrating ornamental, oil, and ornamental values. The complete genome sequence of *S*. *calvescens* plays an important role in the protection, development, and utilization of its resources. However, to date, there is still no complete cp genome that was characterized for *S. calvescens*, even for the genera *Styrax*. Here, we characterized the complete cp genome sequence of *S. calvescens* (GeneBank accession number: MN560141) based on Illumina pair-end sequencing to provide a valuable complete cp genomic resource.

Total genomic DNA was isolated from fresh leaves of *S. calvescens* habitated in Fuxi (30.03938 N, 118.09422E, Alt. 494 m) in Huangshan, Anhui, China. The voucher specimen was deposited at the herbarium of Nanjing Forestry University (accession number NF2019468). The whole genome sequencing was carried out on Illumina Hiseq platform by Nanjing Genepioneer Biotechnology Inc. (Nanjing, China). The original reading was filtered by CLC Genomics Workbench v9 and the clean reading was assembled into chloroplast genome with SPAdes (Bankevich et al. 2012). Finally, CpGAVAS (Liu et al. 2012) was used to annotate the gene structure and OGDRAW (Lohse et al. 2013) was used to generate the physical map. Based on the Neighbor Joining (NJ), the phylogenetic tree was deduced by MAFFT (Katoh and Standley 2013) and MEGA version 7 (Kumar et al. 2016).

The circular genome of *S. calvescens* was 157,951 bp in size and contained 2 inverted repeat (IRa and IRb) regions of 26,048 bp, which were separated by a large single copy (LSC) region of 87,566 bp, and a small single copy (SSC) region of 18,289 bp. A total of 133 genes are encoded, including 88 protein-coding genes (81 PCG species), 37 tRNAs gene (30 tRNA species), and 8 rRNA genes (4 rRNA species). Most of the genes occurred in a single copy; however, 7 protein-coding genes (*ndhB, rpl2, rpl23, rps12, rps7, ycf2, and ycf15*), 7 tRNA genes (*trnA-UGC, trnI-CAU, trnI-GAU, trnL-CAA, trnN-GUU, trnR-ACG, and trnV-GAC*), and 4 rRNA genes (*4.5S, 5S, 16S, and 23S*) are totally duplicated. A total of 9 protein-coding genes (*atpF, ndhA, ndhB, petB, petD, rpl16, rpoC1, rps16, and rpl2*) contained one intron while the other 3 genes (*clpP, ycf3, rps12*) had 2 introns each. The overall GC content of *S. calvescens* genome is 36.95% and the corresponding values in LSC, SSC, and IR regions are 34.78, 30.28, and 42.92%, respectively.

The phylogenetic analysis was conducted based on 24 Styracaceae cp genomes and 3 taxa (Symplocaceae, Actinidiaceae, and Theaceae) as outgroups with sequenced cp genomes. We found that *S. calvescens* was clustered with other families of Ericales with 100% boot-strap values (Figure 1). In addition, *S. calvescens* was highly supported to be a sister species to *Styrax grandiflorus* in Styracaceae. Furthermore, this study will pave the way for future research to understand the genomic information of the chloroplasts of the Styracaceae and this chloroplast resource could be utilized on the phylogeny, DNA barcoding, and conservation genetics.

**Figure 1.**
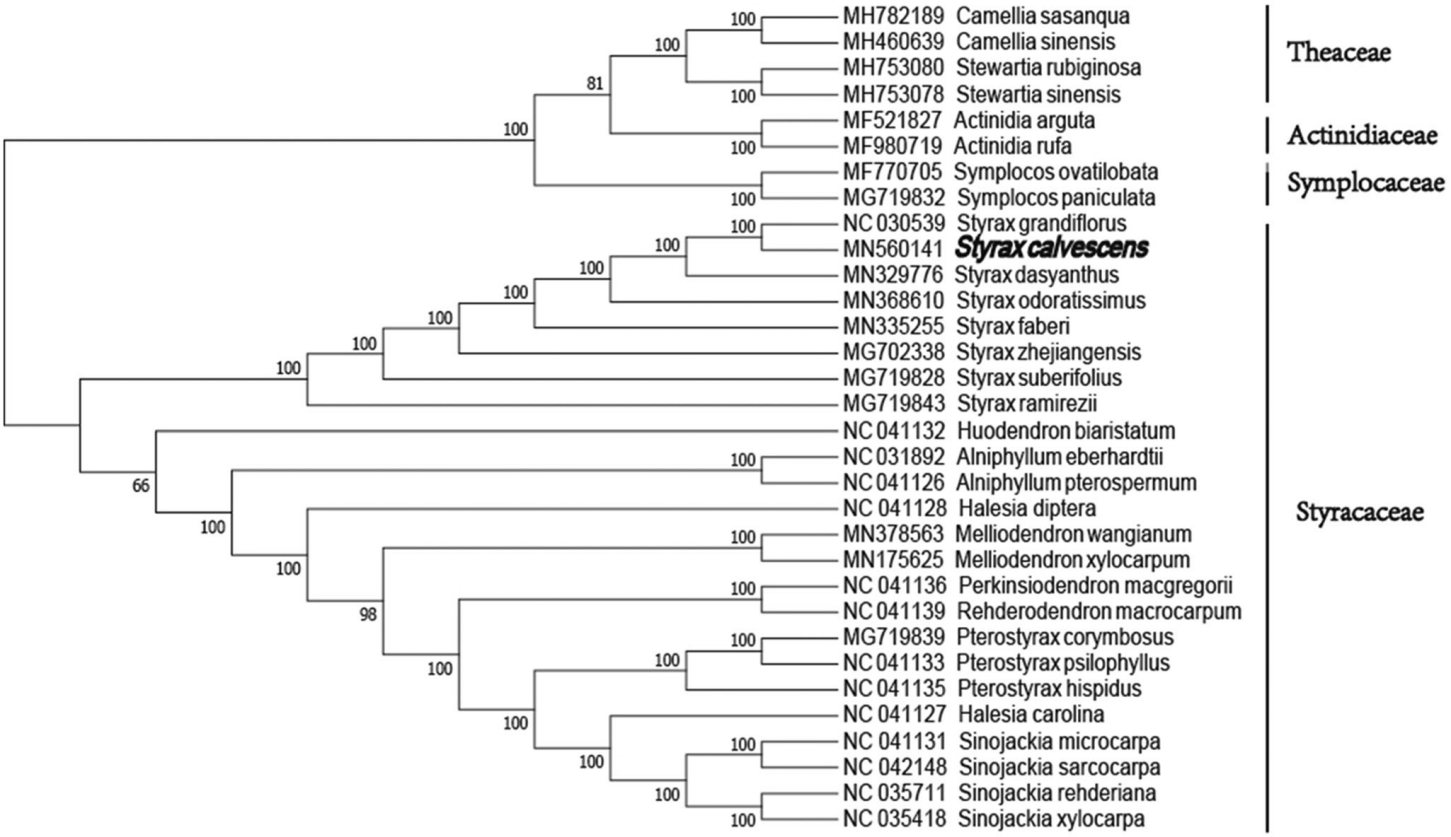
Neighbor Joining tree showing the relationship among 24 representative species within the Styracaceae, based on whole chloroplast genome sequences, with 8 species from Symplocaceae, Theaceae, Actinidiaceae as outgroup. The bootstrap support values shown at the branches.
